# Caspase-3 activation during apoptosis caused by glutathione–doxorubicin conjugate

**DOI:** 10.1038/sj.bjc.6690414

**Published:** 1999-05

**Authors:** T Asakura, T Sawai, Y Hashidume, Y Ohkawa, S Yokoyama, K Ohkawa

**Affiliations:** Department of Biochemistry (I), Jikei University School of Medicine, Tokyo, Minato-ku, 105-8461, Japan

**Keywords:** rat hepatoma cell, doxorubicin, caspase-3, DNA fragmentation, apoptosis

## Abstract

Glutathione–doxorubicin (GSH–DXR) effectively induced apoptosis in rat hepatoma cells (AH66) at a lower concentration than DXR. After 24 h of drug treatment, DNA fragmentation of the cells was observed at the concentration of 1.0 μM DXR or 0.01 μM GSH–DXR. Increase in caspase-3 activity and DNA fragmentation were observed within 12 h and 15 h after treatment with either drug. Intracellular caspase-3 activity was increased in a dose-dependent manner after treatment with DXR or GSH–DXR, and caspase-3 activity correlated well with the ability to induce DNA fragmentation. When the cells were treated with either DXR or GSH–DXR for only 6 h, apoptotic DNA degradation and caspase-3 activation occurred 24 h after treatment. DNA fragmentation caused by these drugs was prevented completely by simultaneous treatment with the caspase-3 inhibitor, acetyl–Asp–Glu–Val–Asp-aldehyde (DEVD-CHO), at 10 μM. By contrast, DNA fragmentation was not prevented by the caspase-1 inhibitor, acetyl–Tyr–Val–Ala–Asp–aldehyde (YVAD-CHO), at the same concentration as DEVD-CHO, and caspase-1 was not activated at all by the treatment of AH66 cells with both DXR and GSH–DXR. These results demonstrate that DXR and GSH–DXR induce apoptotic DNA fragmentation via caspase-3 activation, but not via caspase-1 activation, and that GSH–DXR enhances the activation of caspase-3 approximately 100-fold more than DXR. Moreover, the findings suggested that an upstream apoptotic signal that can activate caspase-3 is induced within 6 h by treating AH66 cells with the drug. © 1999 Cancer Research Campaign


*British Journal of Cancer* (1999) **80**(5/6), 711–715
© 1999 Cancer Research Campaign
Article no. bjoc.1998.0414

### Caspase-3 activation during apoptosis caused by glutathione–doxorubicin conjugate

**T Asakura, T Sawai, Y Hashidume, Y Ohkawa, S Yokoyama and K Ohkawa**

Department of Biochemistry (I), Jikei University School of Medicine, 3-25-8 Nishi-shinbashi, Minato-ku, Tokyo 105-8461, Japan

**Summary** Glutathione–doxorubicin (GSH–DXR) effectively induced apoptosis in rat hepatoma cells (AH66) at a lower concentration than DXR. After 24 h of drug treatment, DNA fragmentation of the cells was observed at the concentration of 1.0 μM DXR or 0.01 μM GSH–DXR. Increase in caspase-3 activity and DNA fragmentation were observed within 12 h and 15 h after treatment with either drug. Intracellular caspase-3 activity was increased in a dose-dependent manner after treatment with DXR or GSH–DXR, and caspase-3 activity correlated well with the ability to induce DNA fragmentation. When the cells were treated with either DXR or GSH–DXR for only 6 h, apoptotic DNA degradation and caspase-3 activation occurred 24 h after treatment. DNA fragmentation caused by these drugs was prevented completely by simultaneous treatment with the caspase-3 inhibitor, acetyl–Asp–Glu–Val–Asp-aldehyde (DEVD-CHO), at 10 μM. By contrast, DNA fragmentation was not prevented by the caspase-1 inhibitor, acetyl–Tyr–Val–Ala–Asp–aldehyde (YVAD-CHO), at the same concentration as DEVD-CHO, and caspase-1 was not activated at all by the treatment of AH66 cells with both DXR and GSH–DXR. These results demonstrate that DXR and GSH–DXR induce apoptotic DNA fragmentation via caspase-3 activation, but not via caspase-1 activation, and that GSH–DXR enhances the activation of caspase-3 approximately 100-fold more than DXR. Moreover, the findings suggested that an upstream apoptotic signal that can activate caspase-3 is induced within 6 h by treating AH66 cells with the drug.

**Keywords:** rat hepatoma cell; doxorubicin; caspase-3; DNA fragmentation; apoptosis

Since many chemotherapeutic agents can induce apoptosis in certain cancer cells, apoptosis may play an important role in cancer therapy (Kaufmann, 1989; Evans and Dive, 1993). However, the molecular mechanisms of anticancer drug-induced apoptosis are still unclear.

Several investigators have reported that activation of intracellular protease is a crucial event in apoptosis (Voelkel-Johnson et al, 1995; Wright et al, 1996). More recent studies have revealed that, in a number of cells, apoptosis is induced by the activation of a series of the caspase family of cysteine proteases (Fernandes-Alnemri et al, 1994; Wang et al, 1994; Nicholson et al, 1995). It has been reported that an inhibitor of caspase-1 or caspase-3 prevents doxorubicin (DXR)-induced apoptosis of human myeloid leukaemia U937 cells (Yamashita et al, 1995).

It has been reported that bovine serum albumin (BSA)–DXR, which increased cytotoxicity against several multidrug resistant cell lines, exhibited the toxic activity after degradation of BSA–DXR into small peptide–DXR conjugate (Takahashi et al, 1996) and that cytotoxicity of glutathione (GSH)–DXR showed the most potent cytotoxicity against AH66 cells among DXR coupled to several small peptides, such as glycylglycine, glycylglycylglycine, GSH, oxidized glutathione and BSA (Asakura et al, 1997a). On the other hand, our recent investigations demonstrated that the cysteine residue of the conjugate was important for expression of the cytotoxicity (Asakura et al, 1997a). Moreover, we showed that GSH–DXR inhibited glutathione S-transferase activity, but DXR did not, indicating that inhibition must be an important contribution to the expression of potent cytotoxicity of GSH–DXR against rat hepatoma AH66 cells (Asakura et al, 1997b). In the present study, we investigated participation of the caspase family in the process of GSH–DXR-induced apoptosis in rat hepatoma AH66 cells.

*Received 22 July 1998*
*Revised 12 November 1998*
*Accepted 17 November 1998*

*Correspondence to:* K Ohkawa

### MATERIALS AND METHODS

### Materials

DXR was obtained from Kyowa Hakko Kogyo (Tokyo, Japan). Acetyl–Asp–Glu–Val–Asp–aldehyde (DEVD–CHO), acetyl–Tyr–Val–Ala–Asp–aldehyde (YVAD-CHD), acetyl–Asp–Glu–Val–Asp–α-(4-methyl-coumaryl-7-amide (DEVD-MCA), acetyl–Tyr–Val–Ala–Asp–α-(4-methyl-coumaryl-7-amide (YVAD–MCA) and 7-amino-4-methyl-counmarin (AMC) were purchased from Peptide Instrument (Osaka, Japan). GSH, RNase A, proteinase K and ethidium bromide were obtained from Sigma (St Louis, MO, USA). Dowex 50Wx8, glutaraldehyde and agarose GP-36 were purchased from Nakarai Tesque (Kyoto, Japan). All other chemicals were of analytical grade.

### Cell lines

Rat ascites hepatoma AH66 cells were cultured with RPMI-1640 containing 10% heat inactivated fetal bovine serum (growth medium) under conventional conditions (Ohkawa et al, 1993; Asakura et al, 1997*a,b*).

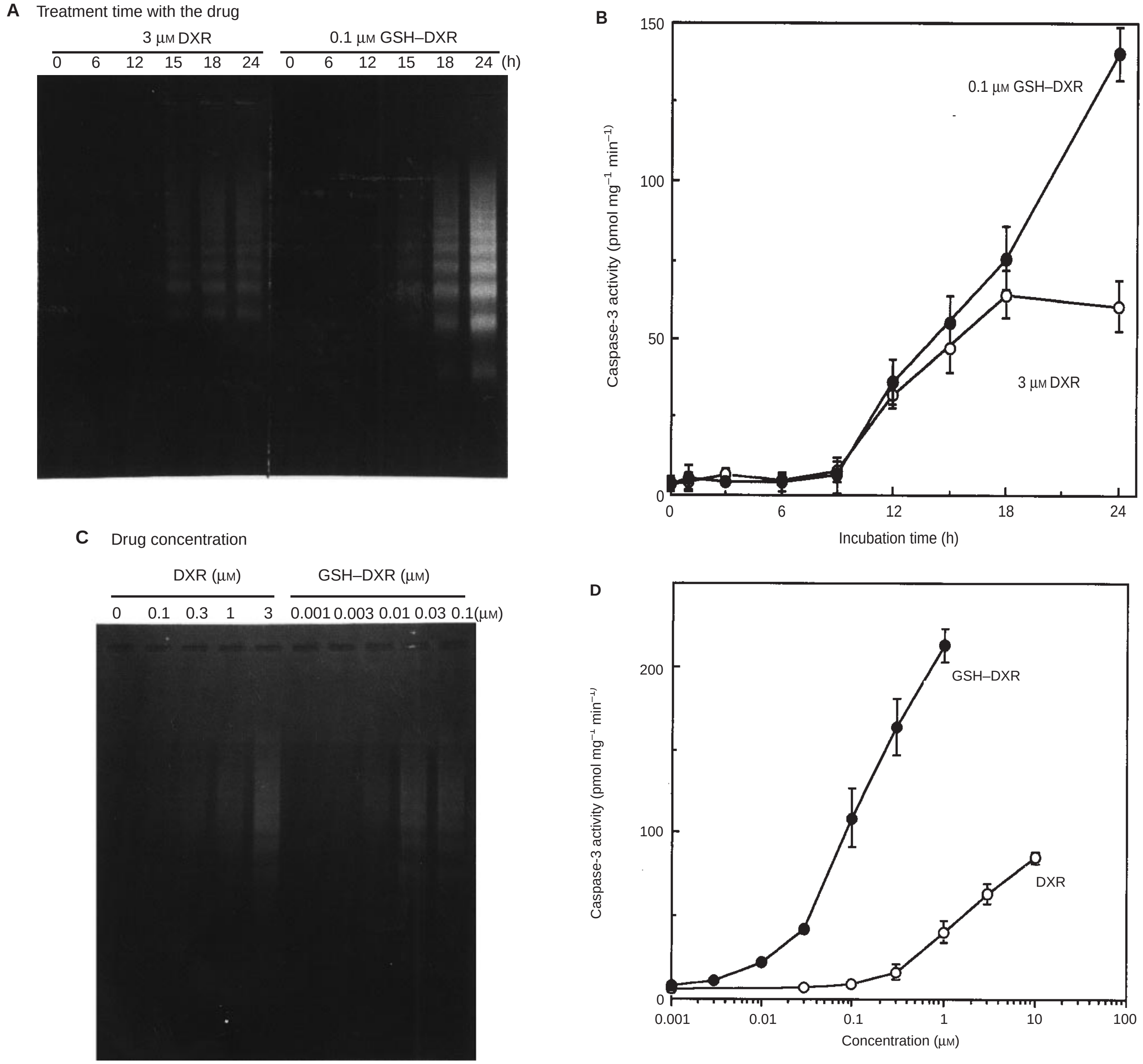

**Figure 1** Induction of DNA fragmentation (**A, C**) and enhancement of caspase-3 proteolytic activity (**B, D**) in AH66 cells treated with DXR or GSH-DXR. (**A, B**) The continuous treatment of the cells with 3 μM DXR (○) or 0.1 μM GSH–DXR (●) for various periods of time. (**C, D**) The continuous treatment of AH66 cells with various concentrations of DXR (○) or GSH–DXR (●) for 24 h. Results are means ± s.d. (three independent experiments)

### Conjugation of DXR with GSH

GSH–DXR was prepared as described previously (Asakura et al, 1997a). In brief, 1 mg of GSH and 0.5 mg of DXR in 0.5 ml of 0.15 M sodium chloride (NaCl) containing 0.1% glutaraldehyde were incubated at room temperature for 30 min. After incubation, GSH–DXR was separated from GSH and DXR using Dowex 50Wx8 ($H^+$ form, 5 × 15 mm). The concentration of DXR was measured by absorbance at 495 nm.

### Preparation of cell extract

After treatment of AH66 cells with DXR or GSH–DXR, harvested cells were washed with ice-cold 0.15 M NaCl and lysed with ice-cold 0.5% Triton X-100 containing 10 mM Tris–HCl (pH 8.0) and 10 mM EDTA. The cell lysate was spun down at 10 000 ***g*** for 10 min and the supernatant was used for the assays of DNA fragmentation and caspase activity.

### DNA fragmentation assay

After treatment of the cells (2 × $10^6$) with DXR or GSH–DXR in the presence or absence of a caspase family inhibitor, the cell extract containing fragmented DNA was incubated with 0.5 mg $ml^{-1}$ RNase A at 37°C for 60 min, then with 0.5 mg $ml^{-1}$ proteinase K at 37°C for 60 min. After incubation, fragmented DNA precipitated by isopropanol was dissolved with 10 mM

 *© Cancer Research Campaign 1999*

Tris–HCl (pH 8.0), 1 mM EDTA, 5% glycerol and 0.05% bromophenol blue. The DNA fragments, separated by 2% agarose gel electrophoresis, were stained with ethidium bromide, and photographed on a UV transilluminator. The 123 base pair DNA ladder (Gibco, BRL, NY, USA) was used as the standard DNA fragments.

### Assay of caspase-3 activity

Reaction mixtures, which contained 100 μM of DEVD-MCA, the appropriate protein concentration of cell extract, 50 mM HEPES–NaOH (pH 7.5), 10% glycerol and 2 mM dithiothreitol with or without 0.1 μM DEVD-CHO, were monitored for AMC liberation at 37°C for 15 min in a spectrofluorometer at an excitation wavelength of 380 nm and an emission wavelength of 460 nm (Nicholson et al, 1995). The caspase-3 proteolytic activity was expressed as the difference between nmol AMC liberations in the presence and absence of the inhibitor per min per mg protein. When the activity of caspase-1 was assayed, 20 μM YVAD-MCA and 0.1 μM YVAD-CHO were substituted for 100 μM DEVD-MCA and 0.1 μM DEVD-CHO in the reaction mixture respectively.

### Protein determination

Protein concentration was assayed by a Bio-Rad protein assay kit (Bio-Rad Lab., Tokyo, Japan) using BSA as the standard.

### RESULTS AND DISCUSSION

### Induction of apoptosis by GSH–DXR

When AH66 cells were continuously exposed to 3 μM DXR or 0.1 μM GSH–DXR for 24 h, the cell viability determined with a colourimetric assay (Ohkawa et al 1993; Asakura et al, 1997*a*, 1997*b*), was decreased to approximately 50% compared with the non-treated cells (data not shown). Inter-nucleosomal DNA fragmentation, a biochemical feature of the apoptotic process, was observed in the cells treated with the drugs. DNA fragmentation occurred within 15 h after continuous treatment with 3 μM DXR or 0.1 μM GSH–DXR (Figure 1A), and concentrations of DXR and GSH–DXR as low as 1.0 and 0.01 μM, respectively, were found to induce DNA fragmentation at 24 h of incubation (Figure 1C). This result indicates that GSH–DXR is a potent inducer of apoptosis as compared with DXR. In our recent report (Asakura et al, 1997*b*), the cytotoxicity of GSH–DXR in AH66 cells was 170-fold higher than that of DXR. Therefore, the extent of cytotoxicity for DXR and GSH–DXR corresponded to the magnitude of apoptosis induced by treatment with these drugs. Moreover, GSH–DXR showed approximately tenfold more cytotoxic activity than other large molecular weight derivatives of DXR, such as DXR conjugated with BSA or with oxidized glutathione against AH66 cells (Asakura et al, 1997*a*). On the other hand, cytotoxicities of DXR coupled to several small peptides, such as glycylglycine and glycylglycylglycine, demonstrated almost the same cytotoxic activity as DXR (Asakura et al, 1997*a*). The magnitude of apoptosis induced by treatment with these conjugates also corresponded to the extent of cytotoxicity for these drugs (data not shown).

### Inhibition of apoptosis by caspase inhibitor

In order to determine the kind of proteases involved in the apoptotic process, the effects of two cysteine protease inhibitors, YVAD-CHO (Thornberry et al, 1992) and DEVD-CHO (Nicholson et al, 1995), on the apoptosis of AH66 cells were determined. As shown in Figure 2A, DEVD-CHO strongly inhibited drug-induced DNA fragmentation in a dose-dependent manner, and 10 μM of the inhibitor completely blocked DNA fragmentation. By contrast, YVAD-CHO (10 μM) did not exhibit any

**A** DNA fragmentation

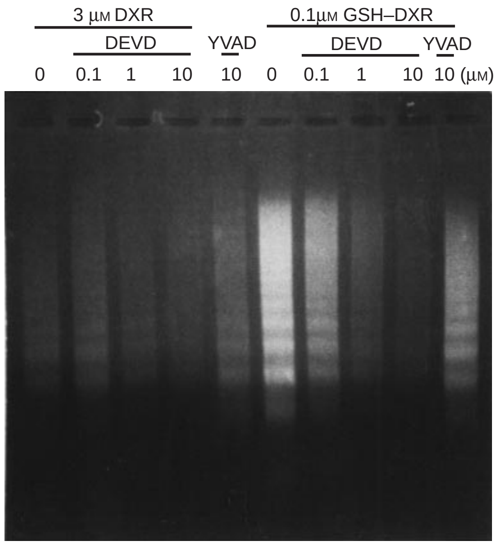

**B** Caspase-3 activity

| Drugs | Caspase-3 activity (pmol mg$^{-1}$ min$^{-1}$) |
|---|---|
| None | 3.2 ± 1.7 |
| 3 μM DXR | 58.7 ± 4.3 |
| + 0.1 μM DEVD-CHO | 41.3 ± 5.5 |
| + 1.0 μM DEVD-CHO | 10.8 ± 3.1 |
| + 10 μM DEVD-CHO | 5.3 ± 1.9 |
| + 10 μM YVAD-CHO | 60.2 ± 7.2 |
| 0.1 μM GSH–DXR | 127.4 ± 10.8 |
| + 0.1 μM DEVD-CHO | 59.6 ± 6.9 |
| + 1.0 μM DEVD-CHO | 33.3 ± 2.7 |
| + 10 μM DEVD-CHO | 7.4 ± 2.0 |
| + 10 μM YVAD-CHO | 120.9 ± 20.4 |

**Figure 2** Prevention of drug-induced apoptosis by caspase inhibitor. AH66 cells were treated with 3 μM DXR or 0.1 μM GSH–DXR, and DEVD-CHO (0.1, 1 or 10 μM) or 10 μM YVAD-CHO, simultanously for 24 h. (**A**) DNA fragmentation. (**B**) Caspase-3 activity in the cells treated with inhibitor. Results are means ± s.d. (three independent experiments)

*© Cancer Research Campaign 1999*

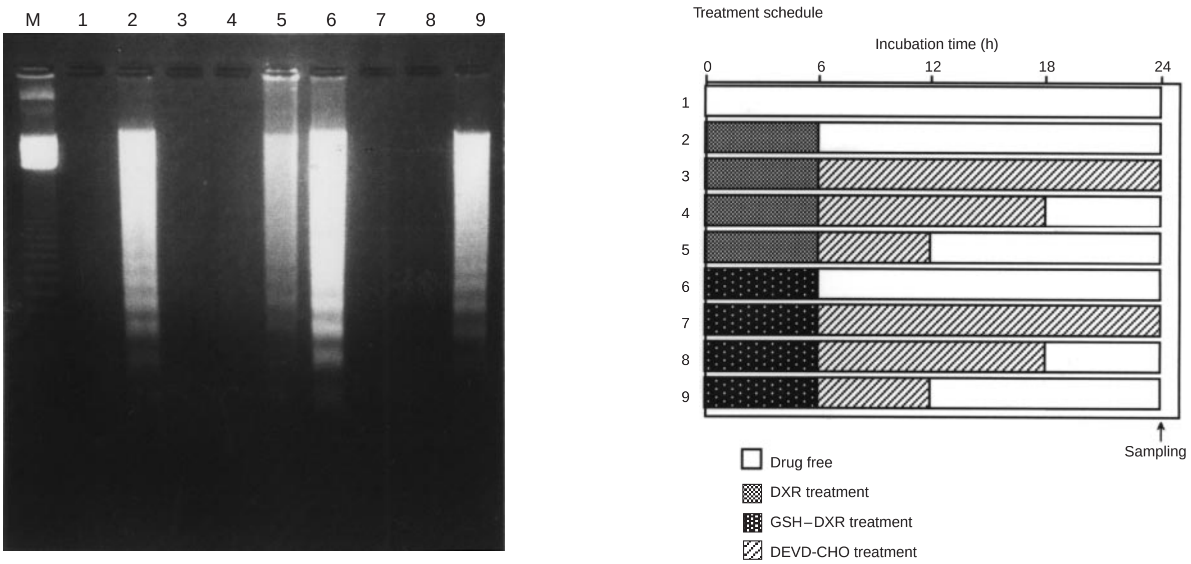

**Figure 3** Prevention of drug-induced DNA fragmentation by delayed treatment of AH66 cells with DEVD-CHO (10 μM) as shown in treatment schedule. Treatment of AH66 cells with 3 μM DXR or 0.1 μM GSH–DXR. M, 123 base pair DNA ladder protective effect on drug-induced apoptosis. This result suggests that DXR- and GSH–DXR-induced DNA fragmentation occur via activation of caspase-3, but not of caspase-1. Several reports have described that anthracycline was able to induce inter-nucleosomal DNA fragmentation in treated cells (Kaufmann et al, 1993; Bose et al, 1995; Yamashita et al, 1995; Chen et al, 1996; Mizushima et al, 1996), but the magnitude of the process induced by GSH–DXR treatment was markedly more potent than that induced by DXR.

### Activation of caspase-3 by treatment with GSH–DXR

When AH66 cells were treated continuously with 3 μM DXR or 0.1 μM GSH–DXR, caspase-3 proteolytic activity in the cells did not increase until 9 h, and increased linearly thereafter to a level approximately 20- or 50-fold higher than that of the non-treated control by 24 h respectively (Figure 1B). By treating with a higher concentration of the drugs (100 μM DXR or 10 μM GSH–DXR), enhancement of caspase-3 activity was not observed until 9 h (data not shown). Therefore, caspase-3 was not activated until 9 h after treatment with the drugs independent of the drug concentration. These drugs increased caspase-3 activity in a dose-dependent manner (Figure 1D). However, caspase-1 proteolytic activity was not increased in cells treated with DXR or GSH–DXR in any time period (data not shown). This result suggests that GSH–DXR enhances the activity of caspase-3 about 100-fold more than DXR-induced activation and the magnitude of the activation induced by treatment with the drugs correlates with the extent of DNA fragmentation.

When AH66 cells were treated simultaneously with DEVD-CHO and the drug (3 μM DXR or 0.1 μM GSH–DXR) for 24 h, cellular caspase-3 activity failed to increase (Figure 2B). However, YVAD-CHO (10 μM) did not affect drug-induced activation of caspase-3. It has been reported that active caspase-3 is generated from its inactive precursor form by other active caspases via Fas (Enari et al, 1996). Therefore, this result suggests that caspase-1 does not participate in proteolytic activation of caspase-3 in drug-induced apoptosis.

### DEVD-CHO prevents drug-induced apoptosis pathway

To examine whether or not DEVD-CHO could inhibit the initial DXR- or GSH–DXR-induced DNA damage or the apoptotic signal pathway itself, the cells were co-treated with the drug and DEVD-CHO in various time schedules (Figure 3). When the cells were treated with DXR or GSH–DXR for 6 h, DNA fragmentation was induced 24 h after the treatment with the drug. It was demonstrated that treatment of AH66 cells with the drug for 6 h was enough to commit the cells to apoptosis (Figure 3, lanes 2 and 6). Although the apoptotic signal was induced by 6-h treatment with DXR and GSH–DXR, caspase-3 activation and DNA fragmentation occurred 12 h and 15 h after treatment respectively (Figure 1 A,B). On the other hand, after treating AH66 cells for 6 h with DXR or GSH–DXR, the addition of 10 μM DEVD-CHO blocked both DNA fragmentation and caspase-3 activation for as long as 24 h (Figure 3, lanes 3 and 8). However, when DEVD-CHO was washed out 12 h after the treatment with the drug, apoptotic DNA degradation occurred 12 h after the wash-out (Figure 3, lanes 5 and 9). This result suggests that DEVD-CHO can inhibit the following apoptotic signal pathway, but does not affect the initial drug-induced DNA damage in AH66 cells. By washing out the inhibitor, caspase-3 activity in the cells was increased as compared with that in the non-treated cells (5.1 to 72.5 pmol $mg^{-1}$ $min^{-1}$). These results indicate that DEVD-CHO does not cause the GSH–DXR-induced apoptotic signal to disappear, but the signal for caspase-3 activation is temporarily suppressed. Moreover, the findings suggested that an upstream apoptotic signal able to activate caspase-3 was already induced by treatment of AH66 cells with DXR or GSH–DXR for 6 h.

GSH–DXR was synthesized by the conjugation between both amino groups of DXR and of GSH via glutaraldehyde. Since the SH group of GSH–DXR determined by fluorescent method using *o*-phthalaldehyde showed the same concentration as DXR, it was demonstrated that the SH group of GSH was present (data not shown). In our recent reports (Asakura et al., 1997*a*, 1997*b*), the SH group on the cysteine of GSH–DXR was important for enhancement of the cytotoxicity, and GSH–DXR inhibited potent

 *© Cancer Research Campaign 1999*

GST activity but DXR did not. On the other hand, it has been proposed that activation of other caspases, or release of granzyme-like substance from granules (Darmon et al, 1995) or cytochrome c from mitochondrial intermembrane (Kluck et al, 1997), or ceramide generation (Bose et al, 1995; Mizushima et al, 1996) induce activation of caspase-3 in cells. It has been reported recently that ceramide links cellular stress responses induced by anticancer drugs, such as DXR, to the CD95 (Apo-1/Fas) pathway of apoptosis in human acute T-cell leukaemia CEM and Jurkat-16 cells (Herr et al, 1997). However, ceramide generation failed to increase in AH66 cells treated with DXR or GSH–DXR in any time period (data not shown). Drug-induced apoptosis in AH66 cells may not be linked to the Fas-mediated pathway.

Further work will attempt to identify the specific signalling molecule and the transducing mechanism that play a role in GSH–DXR-induced apoptosis in AH66 cells.

### REFERENCES

Asakura T, Takahashi N, Takada K, Inoue T and Ohkawa K (1997*a*) Drug conjugate of doxorubicin with glutathione is a potent reverser of multidrug resistance in rat hepatoma cells. *Anti-Cancer Drugs* **8**: 199–203

Asakura T, Ohkawa K, Takahashi N, Takada K, Inoue T and Yokoyama S (1997*b*) Glutathione-doxorubicin conjugate expresses potent cytotoxicity by a suppression of glutathione S-transferase activity: comparison between doxorubicin-sensitive and -resistant rat hepatoma cells. *Br J Cancer* **76**: 1333–1337

Bose R, Verheij M, Haimovits-Friedman A, Scotto K, Fuks Z and Kolesnick R (1995) Ceramide synthase mediates daunorubicin-induced apoptosis: an alternative mechanism for generating death signals. *Cell* **82**: 405–414

Chen Z, Naito M, Mashima T and Tsuruo T (1996) Activation of actin-cleavable interleukin 1-converting enzyme (ICE) family protease CPP-32 during chemotherapeutic agent-induced apoptosis in ovarian carcinoma cells. *Cancer Res* **56**: 5224–5229

Darmon AJ, Nicholson DW and Bleackley RC (1995) Activation of the apoptotic protease CPP32 by cytotoxic T-cell-derived granzyme B. *Nature* **377**: 446–448

Enari M, Talanian RV, Wong WW and Nagata S (1996) Sequential activation of ICE-like and CPP32-like proteases during Fas-mediated apoptosis. *Nature* **380**: 723–726

Evans DL and Dive C (1993) Effects of cisplatin on the induction of apoptosis in proliferating hepatoma cells and nonproliferating immature thymocytes. *Cancer Res* **53**: 2133–2139

Fernandes-Alnemri T, Litwack G and Alnemri ES (1994) CPP32, a novel human apoptotic protein with homology to *Caenorhabditis elegans* cell death protein Ced-3 and mammalian interleukin-1β-converting enzyme. *J Biol Chem* **269**: 30761–30764

Herr I, Wilhelm D, Böhler T, Angel P and Debatin K-M (1997) Activation of CD95 (APO-1/Fas) signaling by ceramide mediates cancer therapy-induced apoptosis. *EMBO J* **16**: 6200–6208

Kaufmann SH (1989) Induction of endonucleolytic DNA cleavage in human acute myelogenous leukemia cells by etoposide, camptothecin, and other cytotoxic anticancer drugs: a cautionary note. *Cancer Res* **49**: 5870–5878

Kaufmann SH, Desnoyers S, Ottaviano Y, Davidson NE and Poirier G (1993) Specific proteolytic cleavage of poly(ADP-ribose) polymerase: an early marker of chemotherapy-induced apoptosis. *Cancer Res* **53**: 3976–3985

Kluck RM, Martin SJ, Hoffman BM, Zhon JS, Green DR and Newmeyer DD (1997) Cytochrome c activation of CPP32-like proteolysis plays a critical role in a Xenopus cell-free apoptosis system. *EMBO J* **16**: 4639–4649

Mizushima N, Koike R, Kohsaka H, Kushi Y, Handa S, Yagita H and Miyasaka N (1996) Ceramide induces apoptosis via CPP32 activation. *FEBS Lett* **395**: 267–271

Nicholson DW, Ali A, Thornberry NA, Vaillancourt JP, Ding CK, Gallant M, Gareau Y, Griffin PR, Labelle M, Lazebnik YA, Munday NA, Raju SM, Smulson ME, Yamin T-T, Yu VL and Miller DK (1995) Identification and inhibition of the ICE/CED-3 protease necessary for mammalian apoptosis. *Nature* **376**: 37–43

Ohkawa K, Hatano T, Tsukada Y and Matsuda M (1993) Chemotherapeutic efficacy of the protein-doxorubicin conjugates on multidrug resistant rat hepatoma cell line in vitro. *Br J Cancer* **67**: 274–278

Takahashi N, Asakura T and Ohkawa K (1996) Pharmacokinetic analysis of protein-conjugated doxorubicin (DXR) and its degraded adducts in DXR-sensitive and -resistant rat hepatoma cells. *Anti cancer Drugs* **7**: 687–696

Thornberry NA, Bull HG, Calaycay JR, Chapman KT, Howard AD, Kotsura MJ, Miller DK, Molineaux SM, Weidner JR, Aunins J, Elliston KO, Ayala JM, Casano FJ, Chin J, Ding GJ-F, Egger LA, Gaffney EP, Limjuco G, Palyha OC, Raju SM, Rolando AM, Salley JP, Yamin T-T, Lee TD, Shively JE, MacCross M, Mumford RA, Schmidt JA and Tocci MJ (1992) A novel heterodimeric cysteine protease is required for interleukin-1β processing in monocytes. *Nature* **356**: 768–772

Voelkel-Johnson C, English AJ, Wold WSM, Gooding LR and Laster SM (1995) Activation of intracellular proteases is an early event in TNF-induced apoptosis. *J Immunol* **154**: 1707–1716

Wang L, Miura M, Bergeron L, Zhi H and Yuan J (1994) Ich-1, an Ice/ced-3-related gene, encodes both positive and negative regulators of programmed cell death. *Cell* **78**: 739–750

Wright SC, Wei QS, Kinder DH and Larrick JW (1996) Biochemical pathways apoptosis: nicotinamide adenine dinucleotide-deficient cells are resistant to tumor necrosis factor or UV light activation of the 24-kD apoptotic protease and DNA fragmentation. *J Exp Med* **183**: 463–471

Yamashita T, Naito M, Kataoka S, Kawai H and Tsuruo T (1995) Aspartate-based inhibitor of interleukin-1β-converting enzyme prevents antitumor agent-induced apoptosis in human myeloid leukemia U937 cells. *Biochem Biophys Res Commun* **209**: 907–915

© Cancer Research Campaign 1999
